# Chronic Pelvic Pain, Quality of Life, and Patient Satisfaction After Robotic Sacrocolpopexy for Pelvic Organ Prolapse

**DOI:** 10.7759/cureus.28095

**Published:** 2022-08-17

**Authors:** Nimesh Patel, Priyansh Faldu, Mohamed Fayed, Hannah Milad, Pradeep Nagaraju

**Affiliations:** 1 Anesthesiology, Henry Ford Health System, Detroit, USA; 2 Medicine, BJ Medical College, Ahmedabad, IND; 3 Anesthesiology, Pain Management, and Perioperative Medicine, Henry Ford Health System, Detroit, USA; 4 Anesthesia and Critical Care, Wayne State University, Detroit, USA; 5 Urology, Beaumont Health, Troy, USA

**Keywords:** suburethral sling, obstetrics hysterectomy, clinical question research, bowel function, bladder function, patient’s satisfaction, quality-of-life, pelvic organ prolapse (pop), chronic pelvic pain syndrome, pelvic pain

## Abstract

Background and objective

When evaluating repair outcomes in robotic sacrocolpopexy (RSC) for the treatment of pelvic organ prolapse (POP), it has become evident that surgeons usually focus on anatomical improvements and neglect equally important parameters of patient satisfaction and quality of life (QoL). Investigating these factors would aid in achieving a more patient-centered approach to treatment. This study aimed to examine QoL and satisfaction outcomes in women after RSC.

Methods

This study analyzed self-reported patient data regarding RSC for POP performed between October 2009 and February 2017 by fellowship-trained urologists in female pelvic medicine and reconstructive surgery. These patients participated in a survey to assess overall satisfaction and QoL, as well as contributing factors, such as changes in bladder and bowel function, vaginal bulge, and vaginal pain on a 7-point Likert scale (ranging from markedly worse to markedly improved). Data were examined using multivariate regression analysis. Positive treatment response was defined as scores of 6 or 7, whereas negative response was defined as scores of 1 to 5.

Results

The response rate was 41% (156/380), and the median age of the participants was 70 years [interquartile range (IQR): 63, 73]. Of note, 98.7% were Caucasian, with 73% currently in a significant relationship. The median duration since RSC was 2.12 years (IQR: 1.2, 3.7). Overall, 93 (66.9%), patients (23.0%), and 123 patients (88.5%) had a positive treatment response for bladder function, bowel function, and vaginal bulge, respectively. Furthermore, 66% of women had improved QoL, 84% reported improved overall satisfaction, and 91.4% stated that they would recommend RSC to a friend. After controlling for significant covariates, results of a multivariate analysis demonstrated positive treatment response for bladder function [odds ratio (OR): 14.6; p < 0.0001], bowel function (OR: 9.72; p = 0.003), and vaginal bulge (OR: 41.7; p < 0.0001), significantly associated with increased odds of having improved QoL, whereas positive treatment response for vaginal bulge (OR: 26.9; p = 0.023) and recommending RSC to a friend (OR: 175; p = 0.0009) were associated with positive overall satisfaction.

Conclusions

Our findings endorse using RSC surgery for patients with POP based on both QoL improvement and overall post-procedure satisfaction perspective. This study may help encourage surgeons and clinicians to employ a surgical modality that incorporates each patient’s unique treatment desires and goals and provide patients with realistic post-procedure goals and expectations regarding treatment.

## Introduction

Pelvic organ prolapse (POP) occurs when the uterus or vagina descends from its normal anatomical position, and it is associated with a lifetime risk of up to 12% for women by the age of 80 years [1-4]. Almost one-third of patients with POP complain about their quality of life (QoL) worsening due to the condition [5]. The POP incidence rate is expected to increase in the next 40 years, with as many as five million women estimated to be affected by 2050, with a reoperation rate of 30% [1,6]. Every year, approximately 200,000 procedures are performed for POP, which is calculated to increase by 46% by 2050 [7-9]. Age, parity, obesity, pelvic floor injury, connective tissue disorders, hysterectomy, estrogen deficiency, intestinal pathologies, chronic pulmonary disease, and genetics have been previously shown to be risk factors for POP [2,10-15]. The treatment modality to repair POP can be either abdominal or vaginal surgery, with the former approach further sub-classified into open, laparoscopic, and robotic. Surgeons usually choose a treatment modality depending on their comfort level and the patient's comorbidities, age, type of reconstructive procedure, POP extent, the extent of obliterative procedures, and potential complications [16,17].

Since its introduction in 2004, robotic sacrocolpopexy (RSC) has been used to treat POP. Over the years, it has become a widespread and popular treatment option for POP [18]. Compared to other modalities, RSC is less invasive and leads to less perioperative blood loss, lower postoperative pain, quicker return to a normal lifestyle, and reduced complications [19]. Past studies of RSC, which have focused on objective outcomes based on POP-Q stage 1 or less, estimate current anatomical cure rates to be 98.6% [17,20]. To date, research on RSC POP repair has primarily focused on anatomical outcomes; however, little research exists regarding the impact of RSC on functional outcomes [21]. Existing research has focused less on patient satisfaction and QoL and more on quantifiable measures, such as pad weight for incontinence and pelvic organ prolapse quantification (POP-Q) measurements for prolapse. Of late, there has been a greater interest in subjective outcomes due to the emergence of a more patient-centered approach to medicine. Conditions such as POP can limit a patient's interpersonal relationships socially, physically, and psychologically [22]. However, the subjective success of the treatment is not clearly defined. Several studies have used validated questionnaires, such as Pelvic Floor Distress Inventory (PFDI), the Pelvic Floor Impact Questionnaire (PFIQ), Pelvic Organ Prolapse/Urinary Incontinence Sexual Function Questionnaire (PISQ), and Urinary Distress Inventory with a view to quantify patient's symptoms and evaluate subjective success.

However, these validated questionnaires are also problematic in many ways. For instance, many questionnaires are too lengthy for patients to complete, and shorter versions often only identify one symptomatic domain. Clinicians may not accurately understand the patient's overall QoL without additional surveys focusing on various potential POP-related symptoms. Furthermore, the reliability of survey responses may become questionable when the patient population drastically changes or an untested surgical modality is used compared to the initially studied population [15].

This study aims to analyze women's overall satisfaction and QoL following RSC surgery to treat POP. In addition, the influence of symptoms on patients' overall QoL and post-surgery satisfaction will also be assessed.

This article was previously presented as a meeting abstract at the Neurology and Urodynamics Conference in February 2018 [23].

## Materials and methods

This retrospective questionnaire-based study was conducted in the Urology Department of Beaumont Hospital in association with the Michigan Institute of Urology in Royal Oak, Michigan. Institutional Review Board (IRB) approval through Beaumont Hospital was obtained before contacting patients who had undergone RSC treatment for POP (IRB approval number: 2016374). Patients with POP underwent RSC via the standard approach involving the Da Vinci surgical system, performed by fellowship-trained urologists in female pelvic medicine and reconstructive surgery. Concomitant robotic hysterectomy or surgical repair was performed when appropriate. All patients met the criteria for symptomatic vaginal bulge necessitating treatment.

Currently living patients who had been 18 years or older at the time of surgery and who underwent RSC between October 2009 and February 2017 were mailed an investigator-created questionnaire to assess self-reported improvements in QoL and overall satisfaction after RSC. A 7-point Likert Scale measured the degree of improvement in patients' subjective health status after undergoing RSC, including bladder function, bowel function, vaginal bulge, pelvic/vaginal pain, QoL, and overall satisfaction (Table 1).

**Table 1:** 7-Point Likert Scale Questionnaire

7-Point Likert Scale	Treatment Response Categories
1 = Markedly Worse	Non-Positive Treatment Response
2 = Moderately Worse	
3 = Mildly Worse	
4 = Same	
5 = Slightly Improved	
6 = Moderately Improved	Positive Treatment Response
7 = Markedly Improved	

Patients were categorized as having a positive treatment response if they indicated that RSC moderately improved or markedly improved their subjective health status. In addition to assessing improvements in subjective health status, the questionnaire further asked patients about demographic information, subsequent surgical history, and whether the patient would recommend RSC to a friend.

Data are presented as medians and interquartile ranges (IQR) for continuous variables, while frequencies and percentages show categorical variables. Odds ratios (OR) with corresponding 95% confidence intervals and p-values were generated using univariate Firth logistic regressions to find significant predictors of positive treatment response in terms of QoL and overall satisfaction. Firth logistic regression was used instead of standard logistic regression due to a high prevalence of patients with positive treatment responses for the outcomes of this study. A p-value <0.05 was considered statistically significant. Data analysis was performed using SAS 9.4 (SAS Institute Inc., Cary, NC). The full questionnaire is available from the authors upon request.

## Results

Of the 380 patients who were mailed the questionnaire, 156 responded and returned the completed questionnaire (response rate: 41%). Of the 156 returned questionnaires, 17 were excluded from the final data analysis due to incomplete data. The final sample size was 139 patients. The median age of the respondents was 70 years (IQR: 63, 73), while the median duration since RSC was 2.1 years (IQR: 1.2 years, 3.7 years). In addition, 16 patients (11.5%) had undergone at least one related surgery between RSC and responding to the survey (Table 2).

**Table 2:** Surgeries post-RSC RSC: robotic sacrocolpopexy

Surgeries	Number of Patients
Hysterectomy	4
Rectocele	3
Enterocele	1
Bowel Obstruction	1
Urethral Sling	1
Mesh Excision	3
Other	3
Total	16

One patient had an entangled bowel following RSC, requiring emergency surgery and ICU admission for nine days. Overall, 93 (66.9%), 32 (23.0%), and 123 patients (88.5%) had a positive treatment response for bladder function, bowel function, and vaginal bulge, respectively. Of the 57 patients who self-reported pre-RSC pelvic/vaginal pain, 35 patients (61.4%) had a positive treatment response post-procedure. Most patients (91.4%) said they would recommend RSC to a friend. Self-reported patient demographics were stratified according to positive treatment response and non-positive treatment response for QoL and overall satisfaction (Table 3).

**Table 3:** Study Variables Stratified by Quality of Life and Overall Patient Satisfaction IQR: interquartile range

		Overall Cohort (n = 139)	Quality of Life Treatment Response				Overall Satisfaction Treatment Response			
			Positive Response (n = 95)	Non-Positive Response (n = 44)	OR (95% CI)	P-value	Positive Response (n = 116)	Non-Positive Response (n = 23)	OR (95% CI)	P-value
Age (Years)										
	Median (IQR)	70.0 (63.0, 73.0)	70.0 (65.0, 74.0)	69.0 (61.5, 73.0)	1.02 (0.97, 1.06)	0.4658	70.0 (65.0, 74.0)	69.0 (59.0, 71.0)	1.04 (0.99, 1.09)	0.1644
Years From Surgery to Survey										
	Median (IQR)	2.1 (1.2, 3.7)	1.9 (1.2, 3.7)	2.8 (1.3, 3.9)	0.84 (0.68, 1.05)	0.1190	2.1 (1.2, 3.5)	2.0 (0.7, 4.6)	0.89 (0.68, 1.15)	0.3578
Bladder Function										
	Positive Treatment Response	93 (66.9%)	81 (85.3%)	12 (27.3%)	14.6 (6.15, 34.8)	< 0.0001	91 (78.5%)	2 (8.7%)	30.8 (7.69, 124)	< 0.0001
	Non-Positive Treatment Response	46 (33.1%)	14 (14.7%)	32 (72.7%)	Reference Group		25 (22.5%)	21 (91.3%)	Reference Group	
Bowel Function										
	Positive Treatment Response	32 (23.0%)	30 (31.6%)	2 (4.6%)	7.92 (2.02, 31.0)	0.0030	32 (27.6%)	0 (0.0%)	18.1 (1.02, 320)	0.0483
	Non-Positive Treatment Response	107 (77.0%)	65 (68.4%)	42 (95.4%)	Reference Group		84 (72.4%)	23 (100.0%)	Reference Group	
Vaginal Bulge										
	Positive Treatment Response	123 (88.5%)	95 (100.0%)	28 (63.6%)	111 (3.90, 999)	0.0016	113 (97.4%)	10 (43.5%)	41.7 (10.6, 164)	< 0.0001
	Non-Positive Treatment Response	16 (11.5%)	0 (0.0%)	16 (36.4%)	Reference Group		3 (2.6%)	13 (56.5%)	Reference Group	
Pelvic/Vaginal Pain (n = 57)										
	Positive Treatment Response	35 (61.4%)	29 (87.9%)	6 (25.0%)	18.7 (4.79, 72.7)	< 0.0001	33 (76.7%)	2 (14.3%)	16.0 (3.39, 75.1)	0.0005
	Non-Positive Treatment Response	22 (38.6%)	4 (12.1%)	18 (75.0%)	Reference Group		10 (23.3%)	12 (85.7%)	Reference Group	
Race of Patient										
	White	138 (99.3%)	94 (98.9%)	44 (100.0%)	1.45 (0.07, 140)	0.8731	115 (99.1%)	23 (100.0%)	0.59 (0.01, 54.1)	0.8202
	Black	1 (0.7%)	1 (1.1%)	0 (0.0%)	Reference Group		1 (0.9%)	0 (0.0%)	Reference Group	
Ethnicity of Patient										
	Hispanic/Latino	3 (2.2%)	3 (3.2%)	0 (0.0%)	3.36 (0.11, 105)	0.4893	3 (2.6%)	0 (0.0%)	1.45 (0.05, 45.8)	0.8327
	Non-Hispanic/Latino	136 (97.8%)	92 (96.8%)	44 (100.0%)	Reference Group		113 (97.4%)	23 (100.0%)	Reference Group	
Current Relationship Status										
	In a Significant Relationship but Not Living Together	3 (2.1%)	3 (3.2%)	0 (0.0%)	3.80 (0.12, 119)	0.4477	3 (2.6%)	0 (0.0%)	1.42 (0.04, 45.3)	0.8432
	Not in a Significant Relationship	39 (28.1%)	29 (30.5%)	10 (22.7%)	1.53 (0.67, 3.49)	0.3155	32 (27.6%)	7 (30.4%)	0.88 (0.34, 2.30)	0.7901
	Living With Spouse/Partner	97 (69.8%)	63 (66.3%)	34 (77.3%)	Reference Group		81 (69.8%)	16 (69.6%)	Reference Group	
Education Level										
	High School or Technical School Graduate	47 (33.8%)	33 (34.7%)	14 (31.8%)	2.31 (0.14, 39.6)	0.5632	39 (33.6%)	8 (34.8%)	4.65 (0.26, 82.1)	0.2941
	Some College	47 (33.8%)	32 (33.7%)	15 (34.1%)	2.10 (0.12, 35.9)	0.6089	38 (32.8%)	9 (39.0%)	4.06 (0.23, 71.0)	0.3379
	College Graduate	22 (15.8%)	17 (17.9%)	5 (11.4%)	3.18 (0.17, 60.2)	0.4401	21 (18.1%)	1 (4.4%)	14.3 (0.56, 369)	0.1080
	Graduate or Professional School	21 (15.1%)	12 (12.6%)	9 (20.4%)	1.32 (0.07, 24.0)	0.8527	17 (14.7%)	4 (17.4%)	3.89 (0.20, 75.7)	0.3695
	Less than High School	2 (1.5%)	1 (1.1%)	1 (2.3%)	Reference Group		1 (0.8%)	1 (4.4%)	Reference Group	
Current Employment Status										
	Employed Full Time	23 (16.6%)	17 (17.9%)	6 (13.6%)	1.18 (1.16, 1.21)	< 0.0001	17 (14.7%)	6 (26.1%)	0.47 (0.16, 1.42)	0.1796
	Employed Part Time	18 (13.0%)	11 (11.6%)	7 (15.9%)	0.67 (0.66, 0.69)	< 0.0001	15 (12.9%)	3 (13.0%)	0.77 (0.20, 2.95)	0.7075
	Home Maker	12 (8.6%)	9 (9.5%)	3 (6.8%)	1.19 (1.16, 1.23)	< 0.0001	10 (8.6%)	2 (8.7%)	0.73 (0.16, 3.49)	0.6976
	Unemployed	1 (0.7%)	0 (0.0%)	1 (2.3%)	0.01 (0.01, 999)	0.3140	1 (0.9%)	0 (0.0%)	0.47 (0.01, 39.3)	0.7350
	Disabled	2 (1.4%)	0 (0.0%)	2 (4.6%)	0.09 (0.08, 0.10)	< 0.0001	2 (1.7%)	0 (0.0%)	0.87 (0.02, 37.8)	0.9442
	Retired	83 (59.7%)	58 (61.0%)	25 (56.8%)	Reference Group		71 (61.2%)	12 (52.2%)	Reference Group	
Recommend to Friend?										
	Yes	127 (91.4%)	93 (97.9%)	34 (77.3%)	51.5 (2.51, 999)	0.0106	115 (99.1%)	12 (52.2%)	175 (8.28, 999)	0.0009
	Maybe/Not Sure/Possibly	3 (2.1%)	2 (2.1%)	1 (2.2%)	31.7 (0.71, 999)	0.0748	0 (0.0%)	9 (39.1%)	11.4 (0.26, 510)	0.2095
	No	9 (6.5%)	0 (0.0%)	9 (20.5%)	Reference Group		1 (0.9%)	2 (8.7%)	Reference Group	
Other Subsequent Surgeries										
	Yes	16 (11.5%)	10 (10.5%)	6 (13.6%)	0.73 (0.25, 2.14)	0.5627	14 (12.1%)	2 (8.7%)	1.22 (0.28, 5.24)	0.7925
	No	123 (88.5%)	85 (89.5%)	38 (86.4%)	Reference Group		102 (87.9%)	21 (91.3%)	Reference Group	

**Figure 1:**
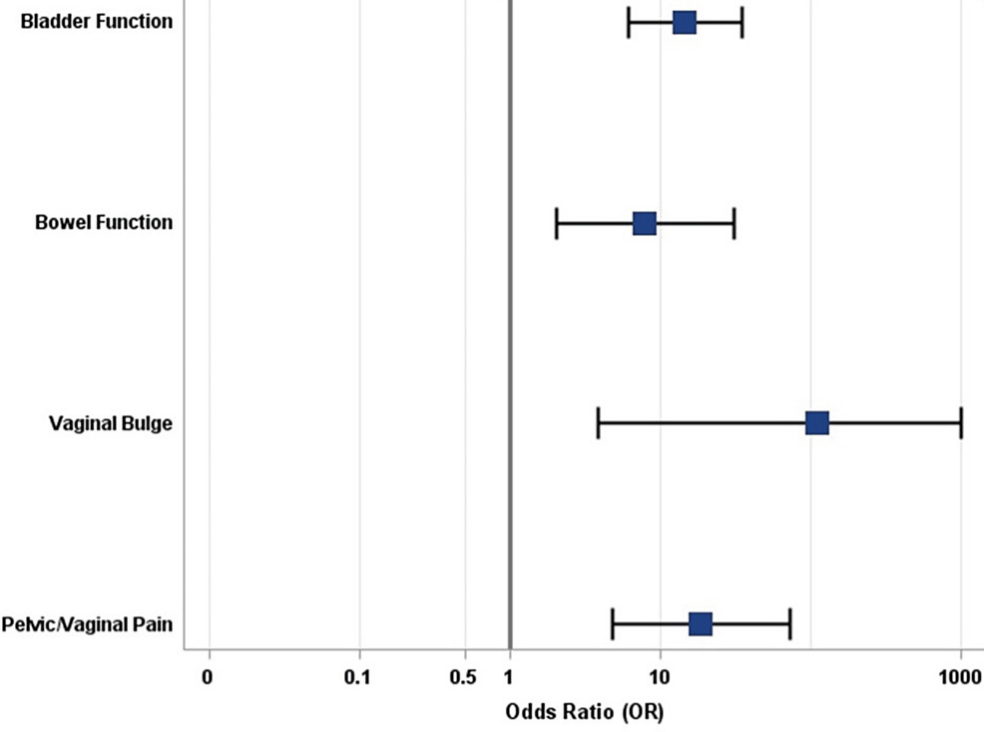
Forest Plot for Positive Treatment Response for Quality of Life

Among patients with positive treatment responses for QoL, 85.3%, 31.6%, and 100.0% of patients also indicated positive treatment responses for bladder function, bowel function, and vaginal bulge, respectively. Univariate logistic regression demonstrated that positive treatment response for bladder function (OR: 14.6; p = < 0.0001), bowel function (OR: 7.92; p = 0.0030), and vaginal bulge (OR: 111; p = 0.0160) were all significantly associated with increased odds of positive treatment response for QoL (Figure 1).

**Figure 2:**
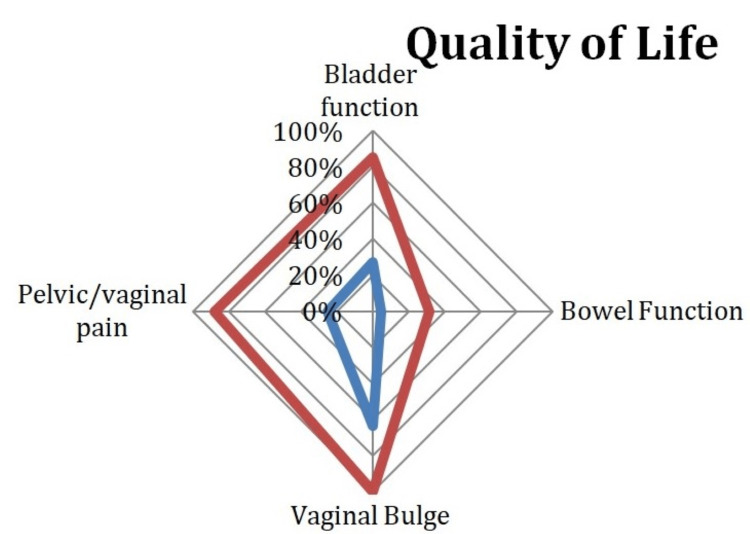
Positive Treatment Response and Non-Positive Treatment Response for Quality of Life Red line: positive treatment response; blue line: non-positive treatment response

Among patients with pelvic/vaginal pain, 87.9% had positive treatment responses for QoL, and this association was also statistically significant (OR: 18.7; p = < 0.0001) (Figure 2).

**Figure 3:**
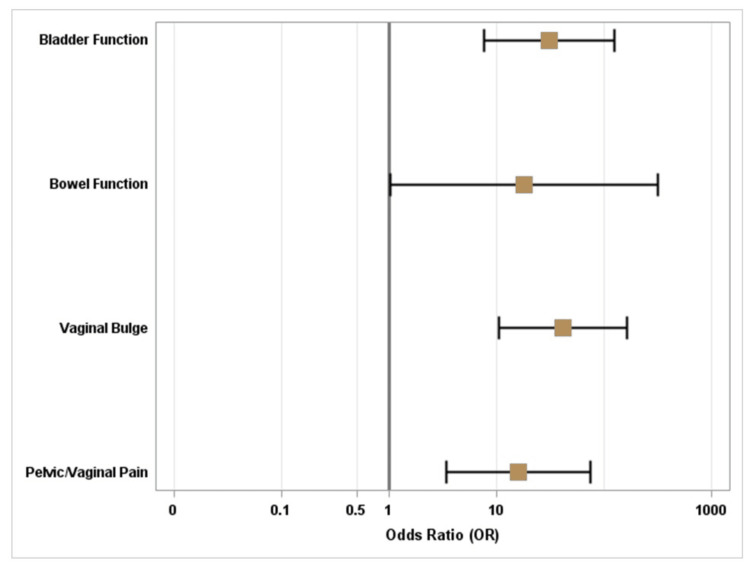
Forest Plot for Positive Treatment Response for Overall Satisfaction

Similarly, 78.5%, 27.6%, and 97.4% of patients with positive treatment responses to bladder function, bowel function, and vaginal bulge, respectively, also had positive treatment responses in terms of overall satisfaction. Positive treatment response for bladder function (OR: 30.8; p = < 0.0001), bowel function (OR: 18.1; p = 0.0483), and vaginal bulge (OR: 41.7; p = 0.0005) were all significantly associated with increased odds of having positive treatment responses in terms of overall satisfaction (Figure 3).

**Figure 4:**
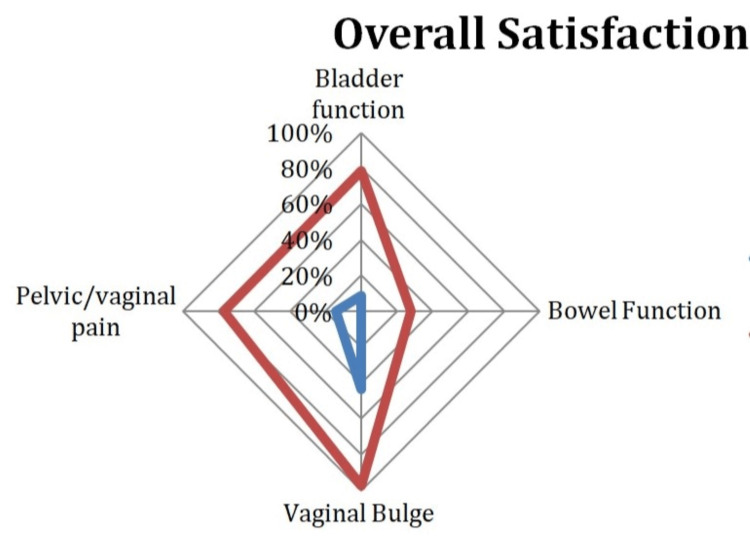
Positive Treatment Response and Non-Positive Treatment Response for Overall Satisfaction Red line: positive treatment response; blue line: non-positive treatment response

For patients who self-reported pelvic/vaginal pain prior to RSC, positive treatment response for pelvic/vaginal pain was also significantly associated with positive treatment response regarding overall satisfaction (OR: 16.0; p = 0.0005), with 76.7% of patients having positive treatment responses for both overall satisfaction and pelvic/vaginal pain (Figure 4).

Current relationship status, education level, and other subsequent surgeries were not associated with positive treatment response for either QoL or overall satisfaction (all p > 0.05). Current employment status was significantly associated with positive treatment response for QoL but not with positive treatment response for overall satisfaction. Patients recommending RSC to friends had significantly increased odds of having positive treatment responses for both QoL and overall satisfaction (p < 0.05).

## Discussion

RSC has come to be routinely used for POP; however, subjective measures of surgical satisfaction, including QoL and overall satisfaction, have yet to be evaluated as a primary aim. Our questionnaire was developed to ascertain patients’ subjective symptoms after RSC and its influence on their QoL and overall satisfaction. POP affects women’s QoL, and it is thus essential to measure QoL when considering a treatment option [24]. QoL was defined as a patient’s overall wellbeing with or without distress from their POP, whereas overall satisfaction pertains to how content patients were with RSC surgery when completing their questionnaire.

One important finding was that positive treatment response for bowel function was significantly associated with positive treatment response for QoL and overall satisfaction. Our results show that positive treatment response for bowel function continues for a median duration of 2.1 years, whereas previously published literature had a median one-year follow-up after RSC surgery [25-27]. In addition, our results show that only 23% of patients reported positive treatment responses for bowel function, whereas the majority had no changes in bowel function. This suggests that RSC surgery dramatically affects the QoL of a minority of patients, who are symptomatic of bowel dysfunction caused by their POP.

Positive treatment response for vaginal bulge significantly influences QoL and overall satisfaction. This finding supports a previous study, which showed that subjective cure, the absence of bulge symptoms, occurred in 92.1% of patients, compared to 88.5% [28]. The improvement in vaginal bulge symptoms is essential to women’s QoL and overall satisfaction, as nearly every patient with positive treatment response in our study also had a positive treatment response for QoL. Likewise, the patient’s pelvic/vaginal pain significantly affects her QoL and overall surgical satisfaction. Previous publications have elucidated that RSC is associated with greater inter- and postoperative pain compared to other surgical modalities [29,30]. However, these findings should be interpreted with caution, since our study indicates that only 38.6% (22/57) of patients did not have a positive treatment response after their RSC surgery, whereas 83% (29/35) with positive treatment response for pelvic pain had improved QoL.

It is well supported within the literature that urinary retention, urinary tract infection, and bladder injury are frequent complications of RSC surgery [31]. Our study shows that these urinary adverse effects should be treated immediately, as bladder function is significantly associated with improving patients’ QoL and overall satisfaction. This is reinforced further by the difference between positive and non-positive treatment responses of 58% and 70% for QoL and overall satisfaction, respectively. This suggests that patients can immediately recognize any bladder problems that negatively affect their QoL or, conversely, improvements in bladder function post-RSC surgery increases QoL and RSC satisfaction.

This study has several limitations that may affect its results and conclusions. Firstly, the investigator-created questionnaire was not validated; however, the investigators could not locate a previously validated survey that adequately examines improvements in subjective health status following this unique procedure. Moreover, the response rate of 41% was low, and we did not draw any conclusions in terms of a comparison between survey responders and non-responders. There is a possibility that patients with favorable outcomes were more likely to complete and return the questionnaire. Finally, due to the high prevalence of positive treatment responses for QoL and overall satisfaction, multivariate logistic regression results were subject to bias from over-fitting and hence were excluded from this analysis.

## Conclusions

Based on our findings, this study endorses using RSC surgery specifically concerning patient QoL improvement and overall satisfaction. An improvement in bladder and bowel function, vaginal bulge, and pelvic pain underscores its contribution to improving patients’ QoL. This study may help surgeons to employ a surgical modality that incorporates the patient’s treatment desires. The results of this study could also help surgeons better understand the factors contributing to satisfaction and QoL to help patients set realistic goals for treatment.
